# Response of Plant-Associated Microbiome to Plant Root Colonization by Exogenous Bacterial Endophyte in Perennial Crops

**DOI:** 10.3389/fmicb.2022.863946

**Published:** 2022-04-05

**Authors:** Svetlana N. Yurgel, Nivethika Ajeethan, Andrei Smertenko

**Affiliations:** ^1^Grain Legume Genetics and Physiology Research Unit, U.S. Department of Agriculture (USDA), Agricultural Research Service (ARS), Prosser, WA, United States; ^2^Department of Plant, Food and Environmental Sciences, Faculty of Agriculture, Dalhousie University, Truro, NS, Canada; ^3^Department of Biosystems Technology, Faculty of Technology, University of Jaffna, Kilinochchi, Sri Lanka; ^4^Institute of Biological Chemistry, Washington State University, Pullman, WA, United States

**Keywords:** microbiome, endophyte, perennial crops, root colonization, microbial cooperation

## Abstract

The application of bacterial inoculums for improving plant growth and production is an important component of sustainable agriculture. However, the efficiency of perennial crop inoculums depends on the ability of the introduced endophytes to exert an impact on the host-plant over an extended period of time. This impact might be evaluated by the response of plant-associated microbiome to the inoculation. In this study, we monitored the effect of a single bacterial strain inoculation on the diversity, structure, and cooperation in plant-associated microbiome over 1-year period. An endophyte (RF67) isolated from *Vaccinium angustifolium* (wild blueberry) roots and annotated as *Rhizobium* was used for the inoculation of 1-year-old *Lonicera caerulea* (Haskap) plants. A significant level of bacterial community perturbation was detected in plant roots after 3 months post-inoculation. About 23% of root-associated community variation was correlated with an application of the inoculant, which was accompanied by increased cooperation between taxa belonging to Proteobacteria and Actinobacteriota phyla and decreased cooperation between Firmicutes in plant roots. Additionally, a decrease in bacterial Shannon diversity and an increase in the relative abundances of *Rhizobiaceae* and *Enterobacteriaceae* were detected in the roots of inoculated plants relative to the non-inoculated control. A strong effect of the inoculation on the bacterial cooperation was also detected after 1 year of plant field growth, whereas no differences in bacterial community composition and also alpha and beta diversities were detected between bacterial communities from inoculated and non-inoculated roots. These findings suggest that while exogenous endophytes might have a short-term effect on the root microbiome structure and composition, they can boost cooperation between plant-growth-promoting endophytes, which can exist for the extended period of time providing the host-plant with long-lasting beneficial effects.

## Introduction

Endophytes can influence plant production by improving plant growth and resistance to biotic and abiotic stresses (Bulgarelli et al., 2013; Busby et al., 2016). Plant microbial endophylism is a widespread relationship that often provides mutual benefits for both micro- and macro-symbionts by directly supplying microbial metabolites to the host-plants or stimulating specific plant responses, which leads to increased enzymatic catalysis and defense responses, and also enhancing nutrients and water uptake (Brader et al., 2014; Chaudhry et al., 2021). Additionally, some endophytic microorganisms can outcompete phytopathogens by occupying the same ecological niche and preventing or decreasing disease occurrence in plants. This type of endophytes can be used as biofertilizers or biocontrol agents (BCAs) to reduce the use of fertilizers and pesticides in agricultural systems that include the production of perennial crops (Compant et al., 2013; Carvalho et al., 2016; Mercado-Blanco et al., 2018; Santos et al., 2019). For example, several plant-associated bacteria and fungi were found to have a mitigation effect on tree diseases, such as canker (TF223, 2020; Shuttleworth, 2021), apple scab (Köhl et al., 2015), and replant diseases (Duan et al., 2021; Wang H.W. et al., 2021). Moreover, the application of exogenous synthetic communities composed of naturally occurring, highly abundant plant-bacteria, becomes a new approach to increased biomass and enhanced root system development in agricultural crops (de Souza et al., 2016; Armanhi et al., 2017, 2021). However, achieving the full benefit of the application of BCAs, it is important to understand the extent of ecological effect of the introduction of exogenous microorganisms into environments and its impact on the host-plant over an extended period of time.

How plant and soil microbiomes respond to an introduction of exogenous microorganisms is a fundamental question in microbial ecology. It is especially important in light of the increasing use of microbe-based supplements in modern agriculture (Santos et al., 2019). There are a few reports addressing this question. For example, it was shown that inoculation with exogenous microbes can improve leguminous crop nodulation and nitrogen fixation (Marek-Kozaczuk and Skorupska, 2001; Vessey and Buss, 2002; Masciarelli et al., 2014; Korir et al., 2017; Puente et al., 2019; Xie et al., 2019). This improved nodulation was correlated with an increased abundance of nitrogen-fixing bacteria in rhizosphere soil, which can be partially explained by an alteration of plants and metabolism reflected in root exudates addition (Xie et al., 2019; Wang H. et al., 2021). Furthermore, it was reported that exogenous arbuscular mycorrhiza affected microbial community diversity and structure (Smith and Read, 2010; Kohler et al., 2016; Wu et al., 2021) and the inoculation of *Mimosa pudica* with *Paraburkholderia phymatum* induced significant alterations in the root-associated microbiome (Welmillage et al., 2021).

In this study, we used a single bacterial endophyte inoculation to address the questions: (1) how the inoculation affects plant-associated microbiome, and (2) how long these effects can be persistent in the plant environment. To address the first question, we evaluated the differences in composition, diversity, and cooperation between the microbiomes associated with inoculated and non-inoculated plants after 3 months post-inoculation. Since soil microorganisms provide a foundation for the plant microbiome (Zarraonaindia et al., 2015), we also looked at the response of soil bacteria to the inoculation. To address the second question, we extended our analysis of the root microbiome for another year to detect long-lasting effect of the inoculation.

## Materials and Methods

### Experimental Design

The experiments were performed on Nova Scotia Haskap farm located in Belmont (45°26′04.4″N 63°22′56.5″W). A total of 40 Aurora variety *Lonicera caerulea* plants, also known as blue honeysucklebare, or haskap were used in the experiments. The cuttings were planted 1-gallon pots using peat base soil rich in organic matter on June 10, 2018. Prior to planting, the root samples (around 3 g) of 21 plants were taken for root and rhizosphere microbiome analysis (S0 and R0). A total of 20 soil samples were taken from the pots for soil microbiome analysis (S0). The pots were watered to settle the soil and remove air pockets.

The strain RF67 was isolated from wild blueberry roots (*Vaccinium angustifolium* Ait) (Dussault, 2019) on MM-NH_4_ media (Somerville and Kahn, 1983) and annotated as Rhizobiales based on the 16S rRNA sequencing. The sequence is available in the NCBI GenBank under the accession number OM753896. About 10 ml of inoculum containing 10^8^ CFU/ml of RF67 was introduced into 20 pots and 20 more pots were left without inoculation. The pots were placed in full sun, and nutrition and watering regimes were maintained as needed along with weeding into fall. After 3 months of plant growth, the soil and root samples were taken from each pot. In May 2019, inoculated and non-inoculated plants were randomly planted in the field and labeled for future identification. In September 2019, the root samples from these plants were taken for root microbiome analysis.

### Sample Preparation

The topsoil litter was removed to expose the surface, if necessary. About 5–10 cm depth soil samples were collected using a sterile spatula, placed in sterile bags, and transported to the laboratory on ice. The soil samples were sieved (2 mm) and stored at −80°C for DNA isolation. Around 3–5 g of roots was collected from each plant. The roots were placed in the sterile bags and transported in the laboratory on ice. The root and rhizosphere samples were processed as described previously (Yurgel et al., 2017). About 0.250 g of soil, rhizosphere, and root tissue was set aside for DNA isolation.

### DNA Extraction and Sequencing

DNA extraction was carried out using the PowerSoil DNA Isolation Kit (MO BIO Laboratories, Carlsbad, CA) according to the manufacturer’s protocol. DNA quality and concentration were measured using a NanoDrop 1000 spectrophotometer (Thermo Scientific, Waltham). About 5 μl of DNA sample was sent to the Dalhousie University CGEB-IMR^1^ for V6-V8 16S rRNA (16S) library preparation and sequencing. Samples were multiplexed using a dual-indexing approach and sequenced using an Illumina MiSeq with paired-end 300 + 300 bp reads. All PCR procedures, primers, and Illumina sequencing details were described as mentioned in the study of Comeau et al. (2017). The DNA was sequenced for prokaryotic V6-V8 16S (ACGCGHNRAACCTTACC forward primer, ACGGGCRGTGWGTRCAA reverse primer) (Srinivasan et al., 2015). All sequences generated in this study are available in the NCBI sequence read archive under the accession numbers PRJNA804723, PRJNA804559, and PRJNA804564.

### Sequence Processing

The overlapping paired-end forward and reverse reads were stitched together using PEAR (Zhang et al., 2014) and exported into QIIME2 (Bolyen et al., 2019). The sequences were trimmed of their primers using QIIME2’s Cutadept plug-in (Martin, 2011; Comeau et al., 2017). Low-quality sequences were filtered from the dataset using QIIME2’s q-score-joined function. Using QIIME2’s Deblur plug-in, the sequences were organized into amplicon sequence variants (ASVs)—high-resolution genomic groupings (Amir et al., 2017; Callahan et al., 2017; Comeau et al., 2017). To account for potential MiSeq bleed-through between runs (estimated by Illumina to be less than 0.1%), ASVs which accounted for less than 0.1% of the total sequences were removed. Taxonomic classifications were assigned to the ASV using QIIME2’s naïve-Bayes scikit-learn function, referencing SILVA databases (Quast et al., 2013; Bokulich et al., 2018). Additionally, ASVs assigned to mitochondria and chloroplasts were filtered out (Comeau et al., 2017).

### Data Analysis

QIIME2’s diversity function was used to calculate Shannon indices (alpha diversity) and also UniFrac matrices (beta diversity) (Lozupone et al., 2011; Kim et al., 2017). These UniFrac matrices were then subjected to an ADONIS test through which their values were fitted to linear regression to determine what proportion of variance in community structure could be attributed to the treatment. Non-metric multidimensional scaling (NMDS) of bacterial communities was performed on Bray–Curtis matrices using the Vegan R package (Oksanen et al., 2016). Differential abundances bacterial taxa were determined using ALDEx2 (Fernandes et al., 2014) with Benjamini–Hochberg corrected *p-*value of the Kruskal–Wallis test (*p* < 0.05). The graphics were produced using ggplot2 (Wickham, 2016). The co-occurrence analysis was performed using the Compositionality Corrected by REnormalization and PErmutation (CCREPE) R package (Schwager et al., 2020) with 1,000 bootstrap iterations and default settings. To obtain comparable datasets from each treatment, 14 replicate samples from R1, R2, R3, and R4 datasets were randomly selected. The co-occurrence and co-exclusion patterns in the samples were scored. The results were filtered to remove non-statistically significant relationships. We generated the network based on the strong correlations with *p*-values < 0.01. The networks were visualized with Cytoscape (Shannon et al., 2003) and were represented as graphs with microbial functions as vertices or nodes and the edges as interaction types.

### Visualization of RF67 Root Infection

C-terminal enhanced green fluorescent protein (eGFP) fusion of CspA2-GFP cloned into pK19 mob *sacB* was used to label RF67 as described previously (Ogden et al., 2019). *Arabidopsis thaliana* seeds were surface-sterilized with 70% (v/v) ethanol for 5 min, air-dried, and sown on 1% (w/v) agar plus half-strength Murashige and Skoog’s basal salt medium (#M524, PhytoTech Laboratories), pH 5.7 plates. Following vernalized for 2 days at 4°C, seedling was grown vertically at 22°C with 16-h light–8-h dark at 100 to 140 μE/m^2^. RF67 overnight culture was added on the top of 10-day-old seedlings and incubated for 24 h. Then, roots were rinsed in water, mounted in water, and imaged using Leica SP8 laser confocal scanning microscope equipped with 40 × 1.3NA oil immersion objective, 488 nm excitation, and 494-535 emission range.

### Growth of RF67 Under Different pH

Cells from 2- to 3-day-old plates were resuspended in Min-salt solution to OD600 = 0.5, and the cell suspensions were diluted with Min-salt solution at 1–10, 1–100, 1–1,000, 1–10,000, and 1–100,000 times in a 96-well microplate. Aliquots of these suspensions were then transferred using a sterile bolt replicator on the plates containing solid nutrient agar (Abdulkadir and Waliyu, 2012) with some modifications. More specifically, since RF67 was very sensitive to NaCl, it was removed from the recipe, and the pH for the media was adjusted to 5, 6, 7, or 8. The colony size was scored after 2–5 days. The endophytes isolated from apple roots and annotated as rhizobium, as well as *Sinorhizobium meliloti* strain 1021 (Galibert et al., 2001), were used as controls.

## Results

### The Strain RF67 Is Capable of Establishing Infection Within Plant Roots

**FIGURE 1:**
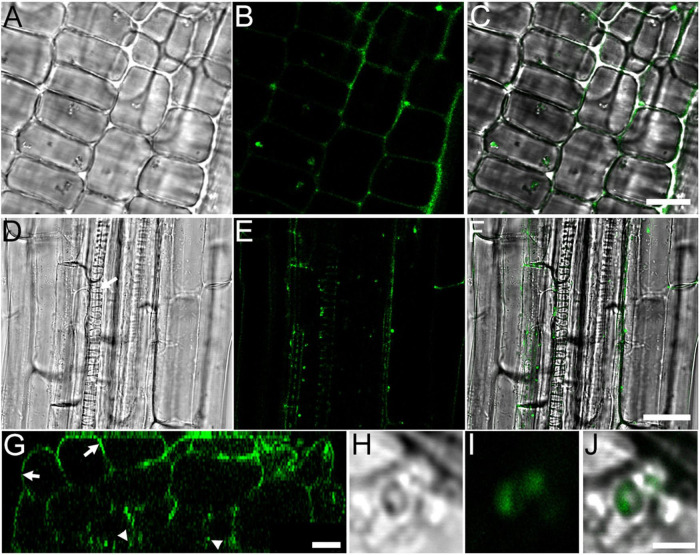
RF67 bacteria colonizes *A. thaliana* roots in 10-days old seedlings. **(A–C)** Localization of bacterial in the apoplast of epidermis cells in root apical meristem. Scale bar is 10 μm. **(D–F)** Localization of bacteria in the apoplast of root differentiation zone. Scale bar is 25 μm. Arrow denotes protoxylem vessel. **(G)** Z-section taken through the stack shown in **(D–F)**. Arrows denote root surface and arrowheads point bacterial cells in the apoplast two to three cell layers below the root surface. Scale bar is 10 μm. **(H–J)** Image of GFP-tagged bacteria in root apoplast. Scale bar is 2 μm. **(A,D,H)** Differential interference contrast (DIC) images; **(B,E,G,I)** GFP fluorescence images; **(C,F,J)** merge of DIC and fluorescence images.

Since RF67 was isolated from wild blueberry root, which usually grows in high acidity soils (Korcak, 1989), we tested the ability of the strain to grow on the plates with a range of pH between 4 and 8. We did not detect any inhibitory effect of pH up to 7 on the growth of strain (Supplementary Figure 1), suggesting its potential tolerance to less acidic environments. To evaluate the ability of RF67 to establish symbiosis with plants, the strain was labeled with GFP protein expressed from *cspA*2 promoter, which is highly active in rhizobia (Ogden et al., 2019). Inoculation of *A. thaliana* roots resulted in the colonization of apoplast of root apical meristem (Figures 1A–C) and in the root differentiation zone where bacterial cells were detected at the root center proximally to xylem cells (Figures 1D–F). Reconstructing transverse (Z-plane) sections (Figure 1G) through the root differentiation zone also confirmed the presence of bacteria three cell layers below the root surface (Figure 1H). Individual bacterial cells in the apoplast were observed under higher digital zoom settings (Figures 1H–J).

### Overall Microbial Community Composition

The dataset retained a total of 12,916 features encompassed by 1,755,900 reads spread across 209 samples, with a mean frequency of 8,401 reads per sample and a median frequency of 5,643 reads per samples. For normalization purposes, the samples were rarefied to a depth of 2,660 reads per sample with total 462,860 reads. This process removed 35 samples with insufficient depth producing 174 samples which included: roots from 21 bare root cuttings (R0); roots from 17 inoculated and 19 non-inoculated plants after 3 months of growth in pots (R1 and R2, respectively); roots from 14 non-inoculated and 15 inoculated plants after 1 year of field growth (R3 and R4, respectively); rhizosphere from 14 bare root cuttings (RS0); rhizosphere form 16 inoculated and 12 non-inoculated plants after 3 months of growth in pots (RS1 and RS2, respectively); 13 samples of soil used for planting (in the time of planting, S0); and 18 inoculated and 15 non-inculcated soil samples from the pots after 3 months of plant growth (S1 and S2, respectively). The final dataset of 12,677 features containing Alphaproteobacteria, Bacteroidia, Gammaproteobacteria, Actinobacterian, and Polyangia as the most abundant bacterial classes represented by 27, 18, 16, 7, and 5% of total microbiome reads, respectively (Supplementary Figure 2).

### Transformation of Microbial Community Over Time

**FIGURE 2:**
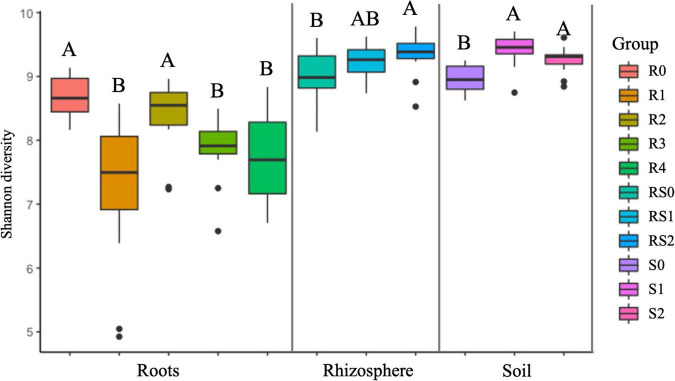
Estimated shannon diversity. For each variable, data followed by different letters are significantly different according to the Kruskal–Wallis pairwise test (*p* < 0.05).

**FIGURE 3:**
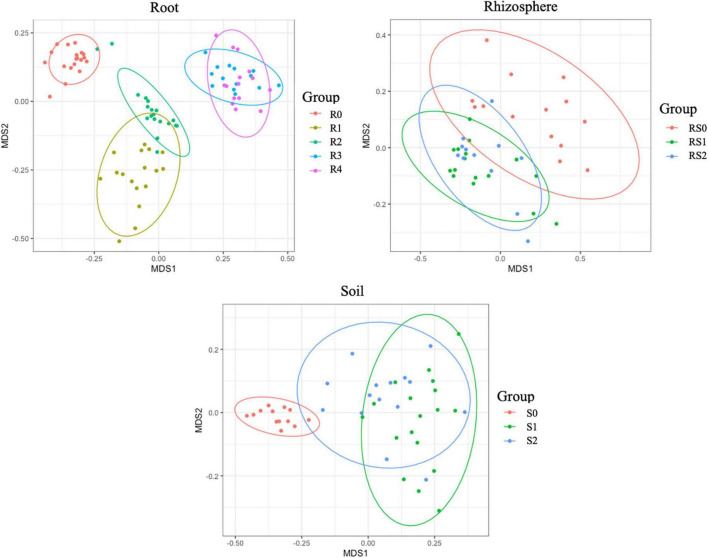
Non-metric multidimensional scaling (NMDS) of bacterial communities. The difference between communities based on Bray–Curtis distances.

**FIGURE 4:**
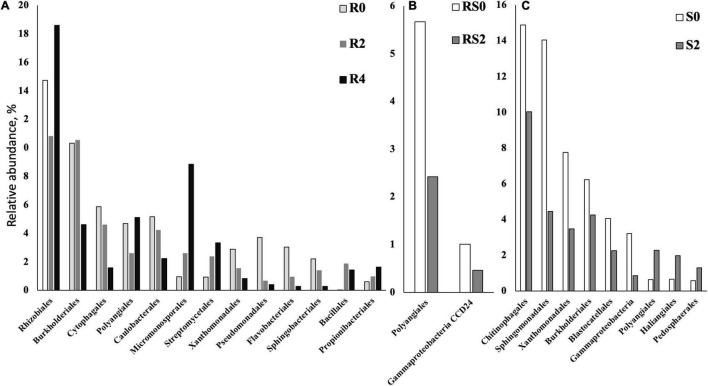
Bacterial taxa that were differentially represented between roots (R), rhizospheres (RS), and soils (S) after different periods of plant growth. Based on the ALDEx2 Benjamini–Hochberg corrected *p*-value of the Kruskal–Wallis test. **(A)** Roots. **(B)** Rhizosphere. **(C)** Soil.

We did not detect significant changes in alpha diversity (Shannon) in the root microbiome at the end of 3-month growth. However, after a year of growth in the field, the Shannon diversity of the microbiome (R4 group) was significantly lower compared to that of R0 and R2 microbiomes (Figure 2). The structure of the root microbiome was visually different when it was grouped based on the time of plant growth (Figure 3), with 47% of community dissimilarity explained by the period of plant growth (R0 vs. R2 vs. R4, *R*^2^ = 0.47, *p* < 0.01; Table 1). We identified 59 bacterial classes differentially represented between the root microbiomes from the plants with different growth stages (Supplementary Table 1). These taxa represented by at least 1% of total 16S reads are shown in Figure 4A. Most notable classes included Rhizobiales and Polyangiales, which were underrepresented in R2 group; and Proteobacteria Burkholderiales, Caulobacterales, Xanthomonadales, and Pseudomonadales, Bacteroidetes Cytophagales and Flavobacteriales, and Sphingobacteriales, which relative abundances decreased over time (Figure 4A). The relative abundances of Actinobacteria Micromonosporales, Streptomycetales, and Propionibacteriales, as well as Bacillales, were increased over time in the root microbiome (Figure 4A).

**TABLE 1:** Variation in sample groupings based on the period of plant growth or inoculation.

Factor	R2
**Period of plant growth**	
R0 × R2 × R4	0.467***
RS0 × RS2	0.267***
S0 × S2	0.492***
**Inoculation**	
R1 × R2	0.225***
R3 × R4	0.057
RS1 × RS2	0.046
S1 × S2	0.138***

Weighted UniFrac beta-diversity distances were calculated for each subset of samples. Adonis tests were used to assess whether beta-diversity is related to sample groupings, 999 permutations, ***p < 0.001.

Rhizosphere microbiome also underwent a significant transformation over the period of first 3-month plant growth, including an increase in Shannon diversity (Figure 2) and the significant dissimilarity in rhizosphere sample grouping (RS0 vs. RS2, *R*^2^ = 0.27, *p* < 0.01; Table 1 and Figure 3). Additionally, bacterial classes Polyangiales and Gammaproteobacteria CCD24 were depleted in the rhizosphere of the plants after 3-month growth compared to the bare root plants (Figure 4B).

After 3-month plant growth, we detected an increase in the bacterial alpha-diversity in the pot’s soil (Figure 2), which was reflected in increased Shannon diversity in S0 compared to the S2 group. Visualization of dissimilarity between soil communities over time revealed a visible trend in beta diversity (Figure 3, S0 vs. S2), and the analysis of strength and statistical significance of sample groupings (ADONIS test) indicated that the time was a significant factor shaping bacterial community (S0 vs. S2, *R*^2^ = 0.50, *p* < 0.01; Table 1). We also detected variations in the relative abundances of several bacterial taxa between initial (S0) and 3-month-old (S2) soils (Figure 4C). More specifically, Chitinophagales, Sphingomonadales, Xanthomonadales, Burkholderiales, Blastocatellales, and Gammaproteobacteria were overrepresented, whereas Polyangiales, Haliangiales, and Pedosphaerales were underrepresented in S0 compared to S2 soils (Figure 4C).

### Distribution of Amplicon Sequence Variant Corresponded to RF67 16S rRNA in Microbiome

We used the RF67 16S rRNA sequence to detect a corresponding ASV, which was identified as 2280c05c4198790e14682350fb738135 and annotated as Alphaproteobacteria *Allorhizobium–Neorhizobium–Pararhizobium–Rhizobium* group. The ASV had a low relative abundance in the non-rarefied ASV table with an average of 0.22 reads per sample. One-way ANOVA showed a significant increase in the number of 2280c05c4198790e14682350fb738135 reads in the R1 group (Tukey, *p* < 0.05; Supplementary Table 2). Additionally, this ASV was detected in S1, RS1, and R3 groups. However, the ASV was also sporadically detected in non-inoculated R0 and R2 groups (Supplementary Table 3). More specifically, 3, 4, 2, and 1 reads corresponding to 2280c05c4198790e14682350fb738135 were detected in samples BF13(R0), BF5Rall(R0), AF22Rall(R2), and AF26Rall(R2), respectively.

### RF67 Inoculation Had a Short-Term Effect on the Diversity and Structure of Root and Bulk Soil Microbiome

**FIGURE 5:**
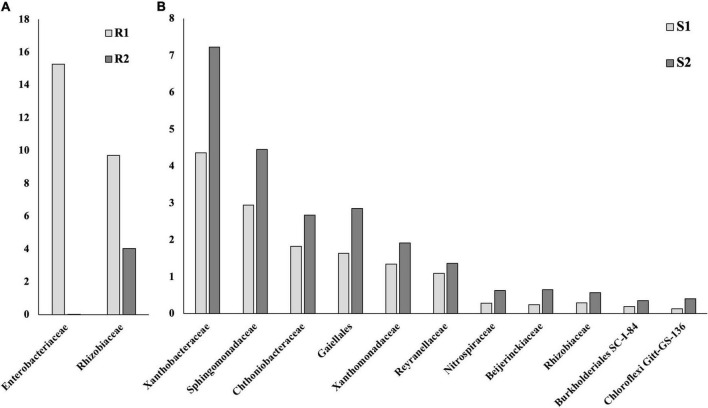
Bacterial taxa that were differentially represented between roots (R), rhizospheres (RS), and soils (S) with and without inoculation. Based on the ALDEx2 Benjamini–Hochberg corrected *p*-value of the Kruskal–Wallis test. **(A)** Roots. **(B)** Soil.

The introduction of RF67 into pots’ soil resulted in the significant decrease of alpha diversity in root microbiome after 3-month plant growth (Figure 2). Shannon diversity in the inoculated roots (R1) was 7.3 vs. 8.4 in the non-inculcated roots (R2). NMDS plots showed a strong visual separation between R1 and R2 groups (Figure 3). This visual community separation was supported by the analysis of strength and statistical significance of sample groupings, indicating that the inoculation was a significant factor shaping bacterial community after 3-month growth (R1 vs. R2, *R*^2^ = 0.23, *p* < 0.01). Additionally, families, such as *Enterobacteriaceae* and *Rhizobiaceae*, were overrepresented in inoculated roots (R1) compared to non-inoculated roots (R2) (Figure 5A).

Bulk soil microbiome responded strongly to the introduction of inoculum. The strength and statistical significance of sample groupings indicated that the inoculation was a significant factor shaping soil microbiome after 3-month growth (S1 vs. S2, *R*^2^ = 0.14, *p* > 0.001) (Table 1), and some visual separations between S1 and S2 groups were detected in NMDS plots. Interestingly, a number of bacterial families had decreased relative abundances in inoculated soils compared to non-inoculated ones, including Alphaproteobacteria *Xanthobacteraceae*, *Sphingomonadaceae*, *Reyranellaceae*, *Beijerinckiaceae*, and *Rhizobiaceae*, Gammaproteobacteria *Xanthomonadaceae* and *Burkholderiales* SC-I-84, Verrucomicrobiae *Chthoniobacteraceae*, Thermoleophilia *Gaiellales*, Nitrospiria *Nitrospiraceae*, and Chloroflexi *Gitt-GS-136* (Figure 5B).

In contrast to the root and bulk soil microbiomes, the inoculation with RF67 did not have detectable effect on rhizosphere microbiome. More specifically, we did not detect any significant differences in bacterial alpha diversity (Figure 2) with an estimated Shannon diversity in the inoculated rhizosphere (RS1) at 9.2 and in the non-inculcated rhizosphere (R2) at 9.3. The strength and statistical significance of sample groupings indicated that the inoculation was not a significant factor shaping rhizosphere microbiome (RS1 vs. RS2, *R*^2^ = 0.05, *p* > 0.05) (Table 1), which was correlated with no visual separation between RS1 and RS2 groups in NMDS plots (Figure 3). Furthermore, no bacterial taxa were differentially represented between RS1 and RS2 groups.

### RF67 Inoculation Had Short-Term Effect on Cooperation Within Root-Associated Community

**FIGURE 6:**
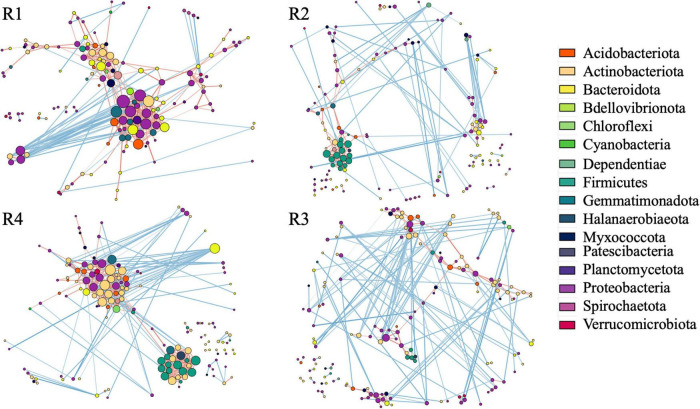
Correlation base network analysis showing potential bacterial interactions. The size of the node is proportional to a taxon’s degree (the number of edges connected to the node). The lines that connect nodes (edges) represent positive (red) or negative (blue) co-occurrence relationship. The intensity of the color and the length of the edges represent the strength of correlation. R1—inculcated roots and R2—non-inoculated roots 3 months post-inoculation, R4—inculcated roots and R3—non-inoculated roots 1 year post-inoculation.

We analyzed the co-occurrence pattern in root microbiomes. Based on the number of taxa associated with clusters representing strong positive interaction, root microbiome from non-inoculated roots exhibited less cooperation compared to inoculated roots (Figure 6). Then, 3 months post-inoculation, network from inoculated roots (R1) comprised of 150 nodes (taxa) with 420 edges (interactions) and maximum node degree (the number of edges connected to the node) 24, compared to 148, 242, and 14, respectively (Supplementary Tables 4, 5). Additionally, in the non-inoculated microbiome (R2), Firmicutes were the major taxa with strong cooperation among them (Supplementary Table 5), whereas Proteobacteria, Bacteroidota, Gemmatimonadota, and Actinobacteriota form a strong cooperation in inoculated roots (Supplementary Table 4).

### RF67 Inoculation Resulted in the Long-Term Increase in Cooperation of Root-Associated Bacteria

After 1 year of field growth, we did not detect any differences in alpha-diversity between inoculated and non-inoculated roots (R3 vs. R4, Figure 2). There was no significant community variation explained by the inoculation (R3 vs. R4, *R*^2^ = 0.06, *p* > 0.05) (Table 1), and NMDS analysis indicated strong visual similarity between the groups. Additionally, no bacterial taxa were differentially represented between inoculated and non-inoculated roots after 1 year of field growth. However, similar to network from 3 months post-inoculated roots, after a year post-inoculation, network from inoculated roots (R4) exhibited much stronger cooperation, compare to non-inoculated roots (R3). More specifically, after a year post-inoculation, R4 network comprised of 155 nodes with 460 edges and maximum node degree 22, compared to 164, 267, and 13 forms R3 network, respectively (Supplementary Tables 6, 7). In the inoculated roots, Actinobacteriota Gaiellales and Acidimicrobiia *IMCC26256*, Bacteroidota *Niastella*, Firmicutes Bacillales, *Planifilum* and *Bacillus thermolactis*, Gemmatimonadota *Gemmatimonas*, Proteobacteria *Pseudolabrys*, and Halanaerobiaeota *Halocella* were the most connected taxa with 17 or more degrees (Supplementary Table 6).

## Discussion

This study examined the response of plant-associated microbiome to the colonization by exogenous bacterial endophyte in perennial crops. However, we started our analysis with evaluation of the transformation of non-inoculated bacterial community over the first 3 months of plant growth, which can provide useful information regarding the dynamic of soil and plant-associated microbiomes during an initial adaptation of the plant to the new environments. Our results indicated significant variations in diversity and structure of bulk and rhizosphere soil and root microbiomes, although these variations did not follow the same pattern in all niches. For example, we detected an increase in bacterial alpha diversity in bulk soils (S2) and rhizosphere (RS2) after 3 months of plant growth, while this parameter was unchanged in root microbiome (R2) in 3 months post-planting and was decreased in the roots of the plants after field growth. This agrees with the previous reports that indicated a decrease in bacterial alpha-diversity along the soil-endosphere continuum (Trivedi et al., 2020). On the other hand, based on the variation in sample groupings and the number of differentially represented taxa between time points, rhizosphere microbiome exhibited more stability over time compared to bulk soil and root microbiomes. This differential dynamic of the microbiomes might be a result of an increased complexity of interactions within microbiomes and plant holobiont (Yurgel et al., 2018).

In the non-inoculated roots, the composition of bacterial community changed over time. While several Proteobacteria taxa significantly decreased, Actinobacteria taxa and Bacillales were increased. It was shown that the plant microbiome is affected by both soil and plant (Bulgarelli et al., 2013; Trivedi et al., 2020). In our study, the changes detected in root microbiome might reflect the changes in soil microbiome during the shift from nursery propagation to growth in the pots and in the fields—as well as the physiological status of the plants—maturation over the 3-month and 1-year period. For example, the profile of soil microbiome also underwent significant changes, including decrease in the relative abundance of Chitinophagales, Sphingomonadales, Xanthomonadales, and unclassified Gammaproteobacteria, the pattern similar to the changes in root microbiome over time. On the other hand, the relative abundances of several Actinobacteria taxa were increased in plant roots over time but not in soils, confirming that the previous studies show that the plant exerts control over its microbiota (Tkacz and Poole, 2015; Trivedi et al., 2020).

The strain RF67 used in our experiments was isolated from roots of perennial crop *Vaccinium angustifolium* and annotated as *Allorhizobium–Neorhizobium–Pararhizobium–Rhizobium* group. There is a substantial scientific evidence showing that rhizobial species can successfully infect and colonize cereal crops (Rosenblueth et al., 2018). To ensure endophytic properties of RF67, we used GFP reporter gene to verify that the strain was able to establish infection and colonization of the host-plant root. Fluorescent bacteria were detected on the root surface and also in the apoplast of both root apical meristem regions where the cell walls are soft and also in the root differentiation zone where the cell walls are harder. Bacterial cells were normally detected two to three cell layers below the root surface. These facts demonstrate the ability of RF67 to penetrate an epidermis cell layer in different root zone and spread through the entire root apoplast. However, bacterial cells were not observed in the cotyledons even after 48 h of co-cultivation. This fact suggests that RF67 can only colonize root tissues.

We also showed, that even though RF67 was isolated from perennial plant preferential to high acidic soils, the strain grew well on the plates with pH 7 typical for Haskap agricultural soils (Iheshiulo et al., 2018) and colonize roots on medium with pH 5.7. Additionally, the initial isolation of the strain RF67 was done on MMNH4 medium with pH 7. This suggests that the strain can survive and grow in the soils used for Haskap cultivation.

Despite the ability of RF67 to establish symbiosis with host-plant and its adaptation to less acidic environment, the ASV corresponding to RF67 was barely detected in inoculated soil, rhizosphere, and root microbiomes. Although it was significantly overrepresented in inoculated roots in 3 months post-inoculation (R1), it was only represented on average by 1.45 reads per sample. We also detected the sporadic presence of RF67 ASV in inoculated rhizosphere and soils after 3 months post-inoculation (RS1), as well as in bare (R0) and non-inoculated roots (R2). To account for potential MiSeq bleed-through between runs, ASV which accounted for less than 0.1% of the total sequences was removed during data processing. However, it is possible that an error in the steps of PCR amplificon during library preparation and sequencing might introduce a single-nucleotide polymorphism, resulting in the false detection of RF67 ASV in the non-inoculated microbiomes. It was recently estimated that the overall observed error rate for samples from the MiSeq platform is 0.473% with standard deviation 0.938 (Stoler and Nekrutenko, 2021). Additionally, a small fragment, such as 16S rRNA V6-V8 region, does not present a determined taxonomic validity (Flores-Felix et al., 2019; Young et al., 2021). This also could explain the fact that these sequences can be found in non-inoculated samples, where other naturally occurring rhizobial spices could be considered.

While we did not detect a high presence of R67 on inoculated microbiomes, the inoculation with RF67 significantly affected in the root and soil microbiomes 3 months post-inoculation. The inoculation induced a significant shift in overall bacterial community structure in the roots and soils and increased bacterial alpha-diversity in the roots. The inoculation also resulted in the increase in the relative abundances of families *Enterobacteriaceae* and *Rhizobiaceae* in the inoculated roots compared to non-inoculated ones. Both these families contain a number of taxa with plant growth promotion capabilities. Interestingly, the inoculation had an opposite effect on the relative abundance of *Rhizobiaceae* in soils. In general, the relative composition of soil microbiome was much stronger affected by inoculation and resulted in the decrease in the relative abundances of a number of bacteria belonging to Alphaproteobacteria and Gammaproteobacteria classes, as well as *Chthoniobacteraceae*, Gaiellales, *Nitrospiraceae*, and Chloroflexi taxa, compared to non-inoculated soil. However, no effect of inculcation was detected in the rhizosphere microbiome’s diversity, composition, and structure. This is consistent with the previous findings which show that root-associated microbial communities were more affected by inoculation compared to the rhizosphere microbiome (Welmillage et al., 2021).

Based on the overall structure of the co-occurrence network, after 3 months post-inoculation (R1), the introduction of RF67 affected the interaction pattern within the root-associated community. The microbiome of the inoculated roots exhibited a stronger cooperation compared to the non-inoculated roots (R2). This was reflected in the 2-fold increase in the number of interactions, compared to non-inoculated roots, and in the formation of a large cluster of strongly cooperating Proteobacteria, Bacteroidota, Gemmatimonadota, and Actinobacteriota in the inoculated roots. In the non-inoculated roots, Firmicutes were the major taxa with strong cooperation among them. When the synthetic community derived from root endophytes was used for inoculation, an increase in the relative abundances of potential plant growth promotion microorganisms was detected (Armanhi et al., 2021). We did not detect a large number of bacterial taxa differentially represented between inoculated and non-inoculated roots, but a number of taxa harboring plant beneficial microbes were among the most connected in the co-occurrence network of inoculated root microbiome. These taxa included *Caulobacteraceae* (Pepe et al., 2013), *Xanthobacteraceae* (Lee et al., 2008), *Gaiellaceae* (Lazcano et al., 2021), *Sphingomonadaceae* (Asaf et al., 2020), and *Chitinophagaceae* (Madhaiyan et al., 2015). These results suggested that the introduction of RF67 boosted cooperation between plant growth-promoting endophytes.

After a year of plant growth, the changes in bacterial community diversity and structure linked to RF67 inoculation were undetectable. The communities from inoculated (R4) and non-inoculated roots (R3) had similar Shannon diversity, as well as no significant effect of inoculation was detected on the significance of sample grouping. However, the co-occurrence network detected strong cooperation between bacteria in the inoculated roots, which was not detected in the non-inoculated roots. Similar to the co-occurrence network from R1, the R4 network had nearly 2-fold increase in the number of interactions, compared to non-inoculated roots. Nevertheless, overtime, the composition of tightly cooperated bacterial had changed. While Proteobacteria, Bacteroidota, Gemmatimonadota, and Actinobacteriota were among the highly connected taxa, a number of Firmicutes also became a part of this group. Interestingly, all the most connected taxa (with the degree at least 17) in R4 co-occurrence network were also found in the co-occurrence networks of R1 and/or R2. This might indicate that after initial strong perturbations in the cooperation between bacteria caused by RF67 inoculation, naturally occurring cooperation (found in R2 but not in R1) began to form in microbiome over time.

## Conclusion

Plant growth-promoting endophytes can be used as BCA to reduce the use of fertilizers and pesticides in agricultural systems including production of perennial crops. The efficiency of these BCA depends not only on the ability of the microorganism to promote plant growth but also on their ability to establish symbiosis with the plant and the stability of the introduced microbes in host-plant tissue over the extended growth periods. In this study, we used a single bacterial inoculation to monitor its effect on the soil and plant-associated microorganisms over a 1-year period. We determined that while bacterial inoculations might have a short-term effect on the composition and structure of soils and root-associated microbiomes, they can boost cooperation between plant growth-promoting endophytes inside the plant roots. We demonstrated that this cooperation could exist for an extended period of time. Therefore, the application of BCA might promote the establishment of symbiosis between naturally occurring plant growth-promoting microorganisms and perennial crops and might provide additional benefits for plant health and production.

## Data Availability Statement

The data presented in the study are deposited in the https://www.ncbi.nlm.nih.gov/genbank/ repository, accession number OM753896 and in the https://www.ncbi.nlm.nih.gov/ repository, accession numbers PRJNA804723, PRJNA804559, and PRJNA804564.

## Author Contributions

SY and AS obtained funding. SY designed the study, collected and processed the samples, wrote the manuscript, and performed data pre-possessing and bioinformatics analyses. AS perform root imaging experiments. NA performed bacterial growth tests. SY, AS, and NA discussed the results and participated in the production and the final version of the manuscript. All authors contributed to the article and approved the submitted version.

## Conflict of Interest

The authors declare that the research was conducted in the absence of any commercial or financial relationships that could be construed as a potential conflict of interest.

## Publisher’s Note

All claims expressed in this article are solely those of the authors and do not necessarily represent those of their affiliated organizations, or those of the publisher, the editors and the reviewers. Any product that may be evaluated in this article, or claim that may be made by its manufacturer, is not guaranteed or endorsed by the publisher.

## Footnotes

1
https://imr.bio
