# Supplementary figures and images for: Response of Plant-Associated Microbiome to Plant Root Colonization by Exogenous Bacterial Endophyte in Perennial Crops
Source: Front Microbiol. 2022 Apr 5;13:863946. doi: 10.3389/fmicb.2022.863946 (PMC9037143; doi:10.3389/fmicb.2022.863946)

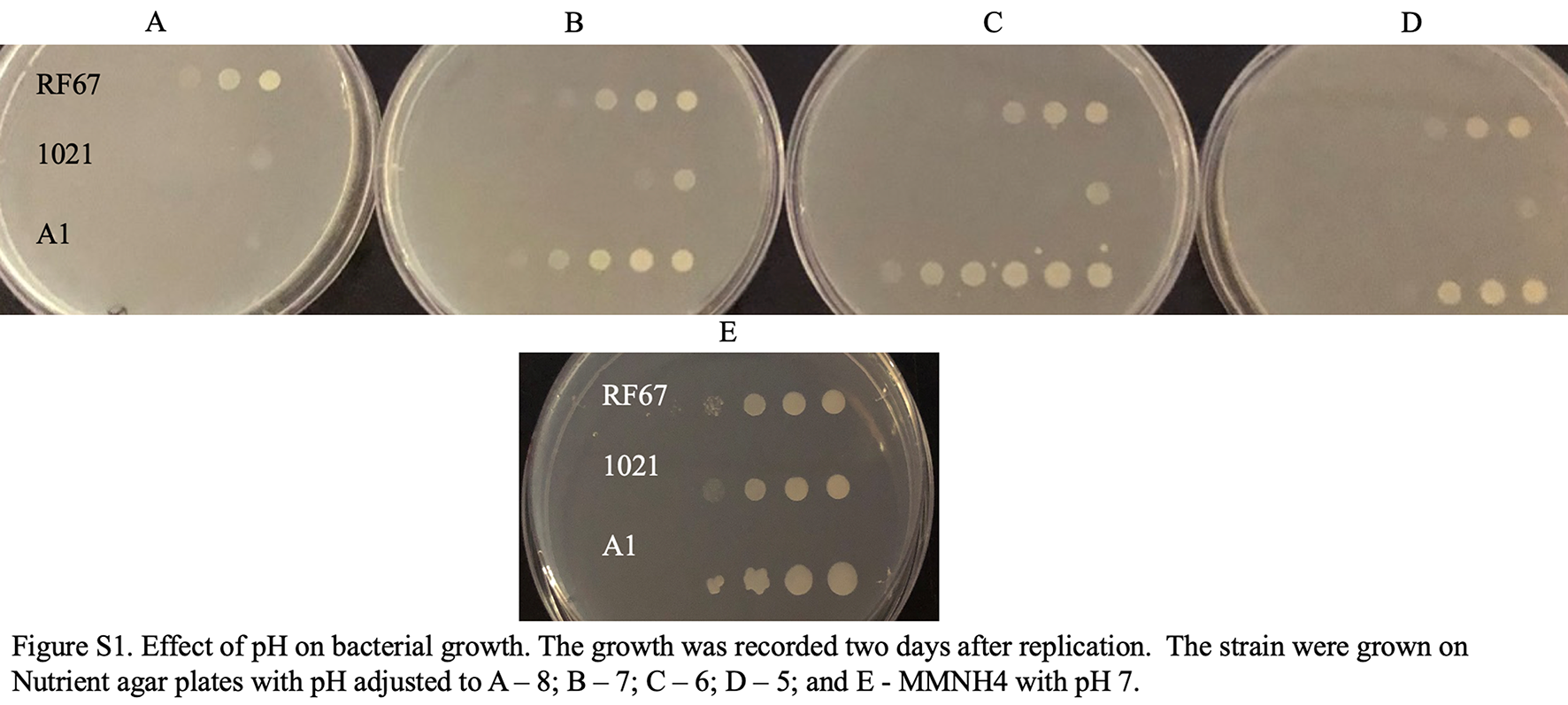

Supplement: Supplementary file 1 [file Image_1.tiff]

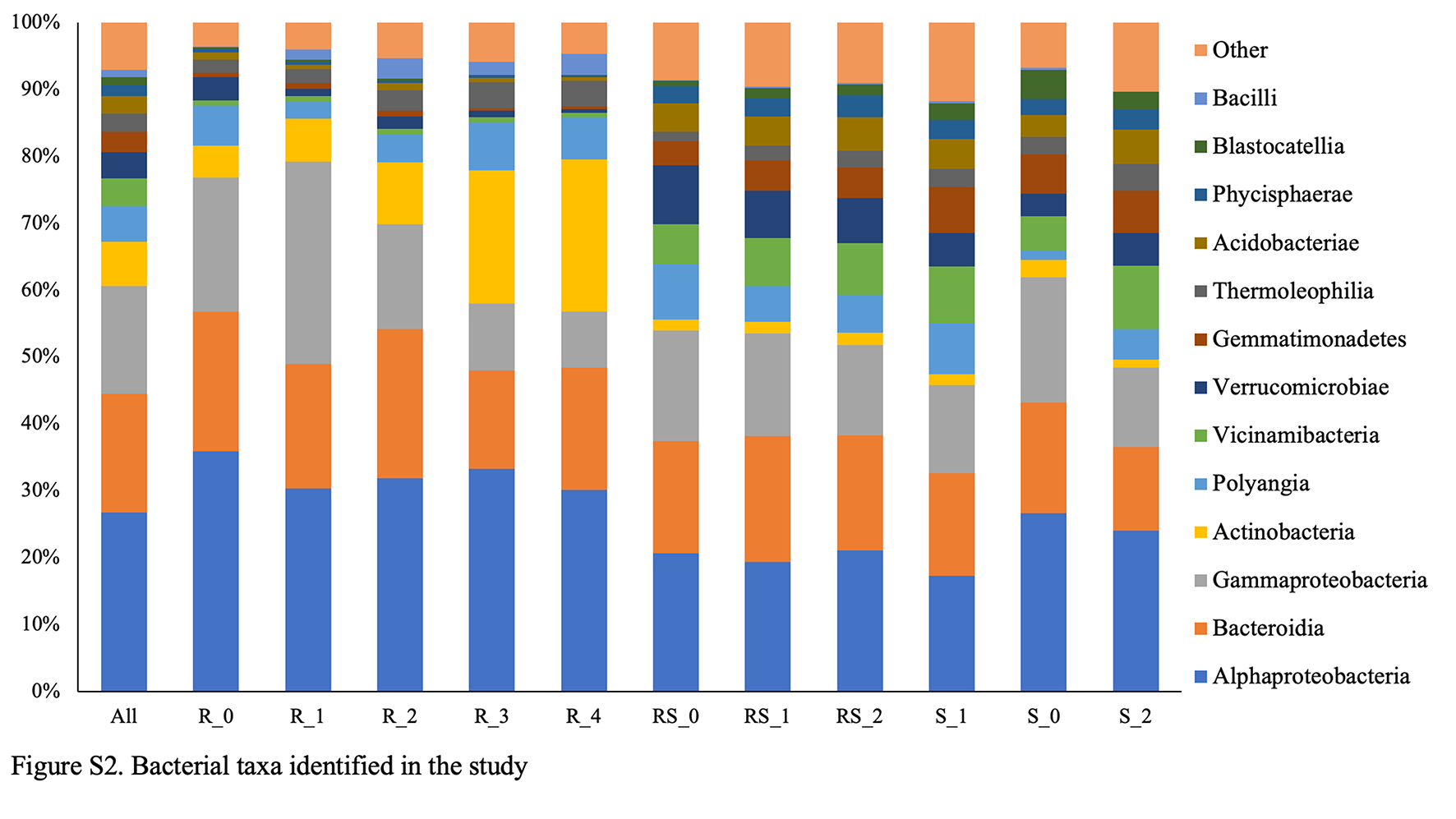

Supplement: Supplementary file 2 [file Image_2.tiff]
