# Supplementary material for: Response of Plant-Associated Microbiome to Plant Root Colonization by Exogenous Bacterial Endophyte in Perennial Crops
Source: Front Microbiol. 2022 Apr 5;13:863946. doi: 10.3389/fmicb.2022.863946 (PMC9037143; doi:10.3389/fmicb.2022.863946)
Supplement: Supplementary file 4 [file Table_2.DOCX]

Table S2. Distribution of ASV corresponding to RF67 in the microbiomes

| **Factor** | **Mean** |
| --- | --- |
| R0 | 0.333 ^B^ |
| R1 | 1.45 ^A^ |
| R2 | 0.15 ^B^ |
| R3 | 0.214 ^B^ |
| R4 | 0 ^B^ |
| RS0 | 0 ^B^ |
| RS1 | 0.111 ^B^ |
| RS2 | 0 ^B^ |
| S0 | 0 ^B^ |
| S1 | 0.0952 ^B^ |
| S2 | 0 ^B^ |

One-way ANOVA, using the Tukey method and 95% confidence. Means that do not share a letter are significantly different. ASV 2280c05c4198790e14682350fb738135
